# The sexist behaviour of immune checkpoint inhibitors in cancer therapy?

**DOI:** 10.18632/oncotarget.22242

**Published:** 2017-11-01

**Authors:** Andrea Botticelli, Concetta Elisa Onesti, Ilaria Zizzari, Bruna Cerbelli, Paolo Sciattella, Mario Occhipinti, Michela Roberto, Francesca Di Pietro, Adriana Bonifacino, Michele Ghidini, Patrizia Vici, Laura Pizzuti, Chiara Napoletano, Lidia Strigari, Giulia D’Amati, Federica Mazzuca, Marianna Nuti, Paolo Marchetti

**Affiliations:** ^1^ Medical Oncology Department, Sant’Andrea Hospital, Rome, Italy; ^2^ Department of Clinical and Molecular Medicine, “Sapienza” University of Rome, Rome, Italy; ^3^ Department of Experimental Medicine, “Sapienza” University of Rome, Rome, Italy; ^4^ Department of Radiological Oncological and Pathological Sciences, “Sapienza” University of Rome, Rome, Italy; ^5^ Statistical Department, “Sapienza” University of Rome, Rome, Italy; ^6^ Breast Diagnosis and Treatment Unit, Sant’Andrea Hospital, “Sapienza” University of Rome, Rome, Italy; ^7^ Oncology Unit, ASST Cremona, Cremona, Italy; ^8^ Division of Medical Oncology 2, IRCCS Regina Elena National Cancer Institute, Rome, Italy; ^9^ Laboratory of Medical Physics and Expert Systems, IRCCS Regina Elena National Cancer Institute, Rome, Italy

**Keywords:** immune checkpoint inhibitors, anti-CTLA-4, anti-PD-1, sex differences, gender differences, Immunology

## Abstract

**Background:**

Immune checkpoint inhibitors, targeting the molecules CTLA-4, PD-1 and PD-L1, showed efficacy against several type of cancers and are currently used in clinical practice. An important biological variable that influences innate and adaptive immunity is the sex, acting through genetic, hormonal and environmental factors. The overall differences between sexes could be crucial to evaluate the response to ICIs.

**Materials and methods:**

We performed a meta-analysis of Phase II-III Clinical Trials published up to June 2017 in which anti-CTLA-4, anti-PD-1 and anti-PD-L1 were studied. We extracted the OS and PFS HR differentiated by sex from subgroups analysis of each trial. We analyzed the three classes of drugs separately.

**Results:**

We selected 36 Phase II-III Clinical Trials, 9 of which reported results for OS and 6 for PFS. We analyzed 2 Clinical Trials for OS with anti-CTLA-4, including 1178 patients, observing a benefit for males vs females (HR 0.65, 95% CI 0.55-0.77 *vs* HR 0.79, 95% CI 0.65-0.96, p 0.078).

Not statistically significant results were observed with anti-PD-1 neither for OS (males *vs* females: HR 0.72, 95% CI 0.64-0.83 *vs* HR 0.81, 95% CI 0.70-0.94, p 0.285) neither for PFS (males *vs* females: HR 0.66, 95% CI 0.52-0.82 vs HR 0.85, 95% CI 0.66-1.09, p 0.158). We cannot perform a meta-analysis for anti-PD-L1 due to the lack of data.

**Conclusions:**

Different mechanisms could be involved in sex differences with regard to immunotherapy. These differences could be relevant to identify immunological targets in order to draw studies exploring novel combinations of immunotherapy agents.

## INTRODUCTION

Several immune checkpoint inhibitors (ICIs), mostly against the molecules CTLA4, PD-1 and PD-L1, are approved in clinical practice, given the promising results in several types of solid tumors. [1] Interestingly, the target of these drugs is not the tumor, as conventional chemotherapy and targeted therapies, but critical checkpoints of the patient’s immune system. In fact CTLA-4 is predominantly expressed on CD4+ “helper” T lymphocytes and its physiological engagement by CD80 and CD86 molecules down-regulates the T cell response. [2] PD-1 is mainly expressed by activated T cells and binds PD-L1, expressed on the surface of antigen presenting cells (APCs) and tumour cells, inducing a negative control damping of the immune response. [2]. These drugs are able to unleash existing T cell, permitting the expansion of effector cytotoxic T cells, that could recognize and destroy the tumor.

Currently, despite the remarkable success also in the metastatic setting of several approved monoclonal antibodies, such as anti-CTLA4 (Ipilimumab, Tremelimumab) and anti-PD1/PD-L1 (Nivolumab, Pembrolizumab, Atezolizumab, Avelumab, Durvalumab), a good percentage of patients do not respond to treatment and may develop different pattern of toxicities, known as immune related adverse events (irAE).

Various biomarkers (e.g. PD-L1 expression, intratumoral immune infiltrate, mutational burden etc...) have been supposed to reflect the ICIs pharmacodynamics and to predict ICIs efficacy and/or toxicity, but their relevance is still unclear. [3].

It was shown that the sex, defined by the differential organization of chromosomes, reproductive organs, and sex steroid levels, represents an important biological variable that influences innate and adaptive immunity. The sex influence on the immune system is well documented in the pathogenesis and prognosis of autoimmune diseases, infections and malignancies [4].

Generally, as showed in various immune-modulated disease, adult females make a stronger innate and adaptive immune responses than males. In particular, females have a higher incidence of autoimmune diseases than males and the onset and the severity is related with the reproductive status, suggesting that their pathogenesis could be influenced by sex hormones. [5, 6].

The different innate immune responses among mammals suggests that some sex differences may be germline encoded. Indeed, in preclinical models of human cells or rats tissue, females show higher expression than males of genes along TLR pathways, like Toll-like receptor 7 (*TLR7*) gene, encoded on the X chromosome, as well as a better induction of type I interferon (IFN) responses. [4, 7-10] Murine models were recently studied to evaluate the different sexual response to ICIs. The treatment with anti-PD-L1 Abs appeared to be more efficacious in female than in male murine model of melanoma. [11]. Interestingly, a recent research reports that PD-1 expression and function correlate with a better response to hormone treatment. [12] Despite of great interest in the improved clinical outcomes of cancer patients with immunological approach, only few data are published regarding potential sex-based differences in responses and toxicity to immunotherapy.

In this context, the overall differences between males and females could be crucial to evaluate the response to ICIs and the toxicity profile.

On these bases, we performed a phase II and III trials’ meta-analysis to determine sex-differential effects of ICIs in cancer patients.

## RESULTS

**Figure 1:**
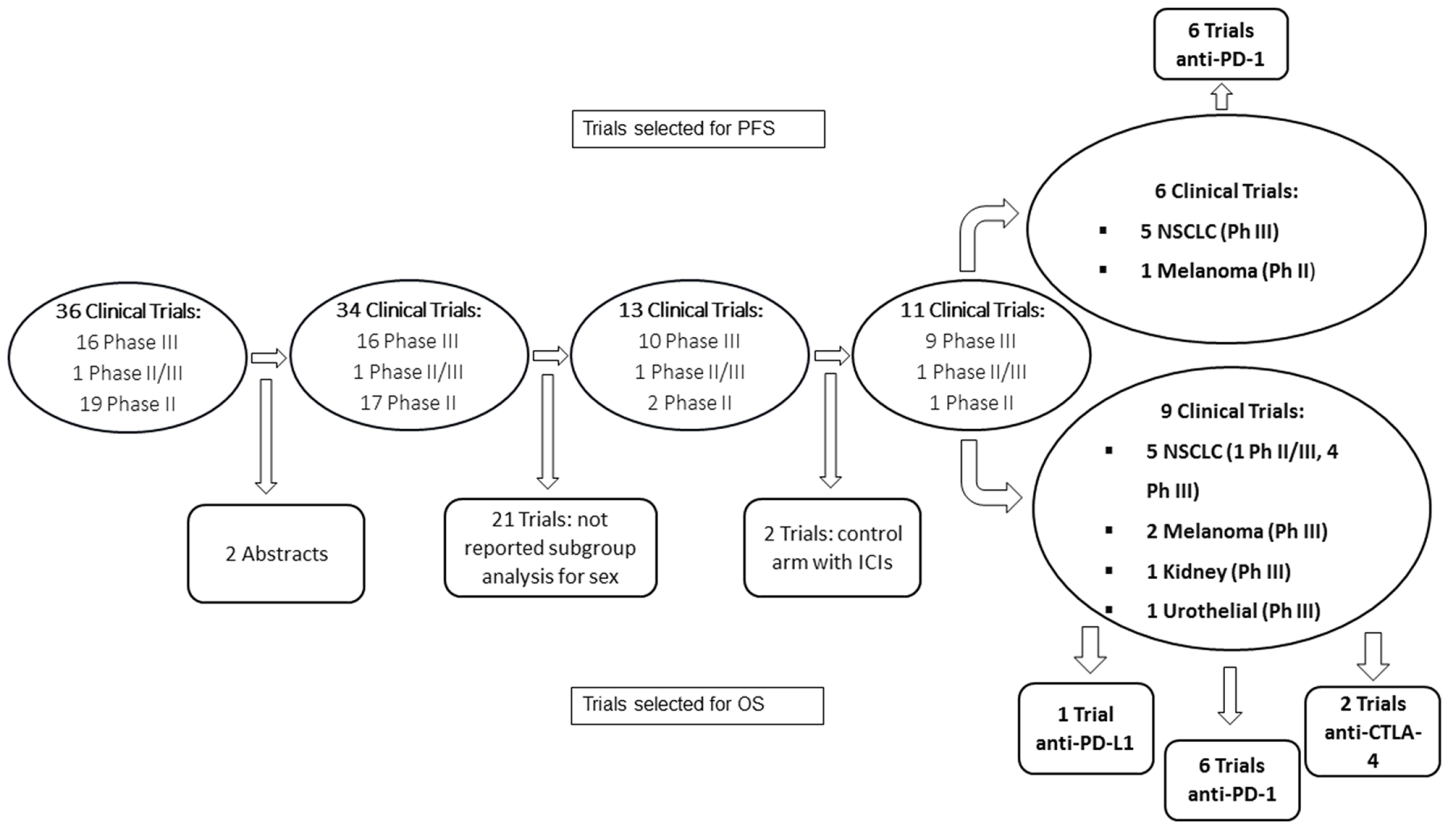
Consort diagram for trial selection

We selected 36 Phase II and Phase III Clinical Trials published up to June 2017 in which immunotherapy was tested (anti-CTLA-4, anti-PD-1 and anti-PD-L1) in patients affected by solid cancers. Of these studies, 17 were Phase III Trials subdivided as following: 8 on melanoma, 6 on NSCLC, 1 on RCC, 1 on head and neck tumors and 1 on urothelial carcinoma. [13-30] Nineteen were Phase II Trials: 10 on melanoma, 5 on NSCLC, 1 on RCC and 3 on urothelial cancer. [31-50] From this selection, 2 studies were excluded because results were published only as abstracts, 21 because the subgroup analysis differentiated by sex was not reported and 2 because the patients in the control arm received ICIs. (Figure 1).

In the final statistical analysis for OS 9 studies were selected as reported in Table 1, including the three classes of drugs. [13, 16, 20, 25-30] The patients enrolled in the standard arm received a platinum doublet chemotherapy in first line or Docetaxel in second line for NSCLC, Everolimus for RCC, Dacarbazine or Gp100 for melanoma, investigator’s choice chemotherapy (Paclitaxel, Docetaxel or Vinflunine) for urothelial cancers. The patients were treated in first or second line, according to the protocol. Only two trials, CheckMate-025 on RCC and Keynote-045 or urothelial carcinoma, enrolled patients in third line of treatment. [29, 30].

**Table 1:** Clinical trials selected for OS

Author/year	Clinical trial	Cancer	Treatment	N pts	M	F	OS HR	OS M HR	OS M range	OS F HR	OS F range
Hodi et al. 2010 [13]	Phase III	Melanoma St. III, IV	Ipilimumab + Gp100	403	247	156	0.68	0.66	0.50-0.87	0.72	0.52-0.99
			Ipilimumab	137	81	56	0.66	0.54	0.37-0.77	0.81	0.55-1.20
			Gp100	136	73	63					
Robert et al. 2011 [16]	Phase III	Melanoma St. IV	Ipilimumab + Dacarbazina	250	152	98	0.72	-0.35^*^	-0.60- -0.1^*^	-0.15^*^	-0.46- -0.16^*^
			Dacarbazina	252	149	103					
Motzer et al. 2015 [29]	Phase III	Kidney St. IV	Nivolumab 3 mg/kg	410	315	95	0.73(0.57-0.93)	0.73	0.58-0.92	0.84	0.57-1.24
			Eveolimus	411	304	107					
Bellmont et al. 2017 [30]	Phase III	Urothelial St. IV	Pembrolizumab 200 mg q21	270	200	70	0.73 (0.59-0.91)	0.73	0.56-0.94	0.78	0.49-1.24
			Chemotherapy	272	202	70					
Brahmer et al. 2015 [26]	Phase III	NSCLC St. IIIB/IV	Nivolumab 3 mg/kg	135	11	24	0.59(0.44-0.79)	0.57	0.41-0.78	0.67	0.36-1.25
			Docetaxel	137	97	40					
Borghaei et al. 2015 [27]	Phase III	NSCLC ADK St. IIIB/IV	Nivolumab 3 mg/kg	292	151	141	0.73 (0.59-0.89)	0.73	0.56-0.96	0.78	0.58-1.04
			Docetaxel	290	168	122					
Carbone et al. 2017 [28]	Phase III	NSCLC St. IV/recurent	Nivolumab 3 mg/kg	271	184	87	1.02 (0.80-1.30)	0.87	0.74-1-.26	1.15	0.79-1.66
			Chemotherapy	270	148	122					
Herbst et al. 2016 [22]	Phase II/III	NCSLC St. IV	Pembrolizumab 2 mg/kg	345	212	133	0.71(0.58-0.88)	0.65	0.52-0.81	0.69	0.51-0.94
			Pembrolizumab 10 mg/kg	346	213	133	0.61 (0.49-0.75)				
			Docetaxel	343	209	134					
Rittmeyer et al. 2017 [25]	Phase III	NSCLC St. IIIB/IV	Atezolizumab 1200 mg	425	261	164	0.73 (0.62-0.87)	0.79	0.64-0.97	0.64	0.49-0.85
			Docetaxel	425	259	166					

*Value expressed as Log HR

We conducted a meta-analysis dividing the selected trials according to the target of the studied drug. We cannot perform a meta-analysis for anti-PD-L1, because only one study was available, showing a HR in females of 0.64 (95% CI 0.49-0.84) vs HR 0.79 (95% CI 0.64-0.97) in males. [25].

**Figure 2:**
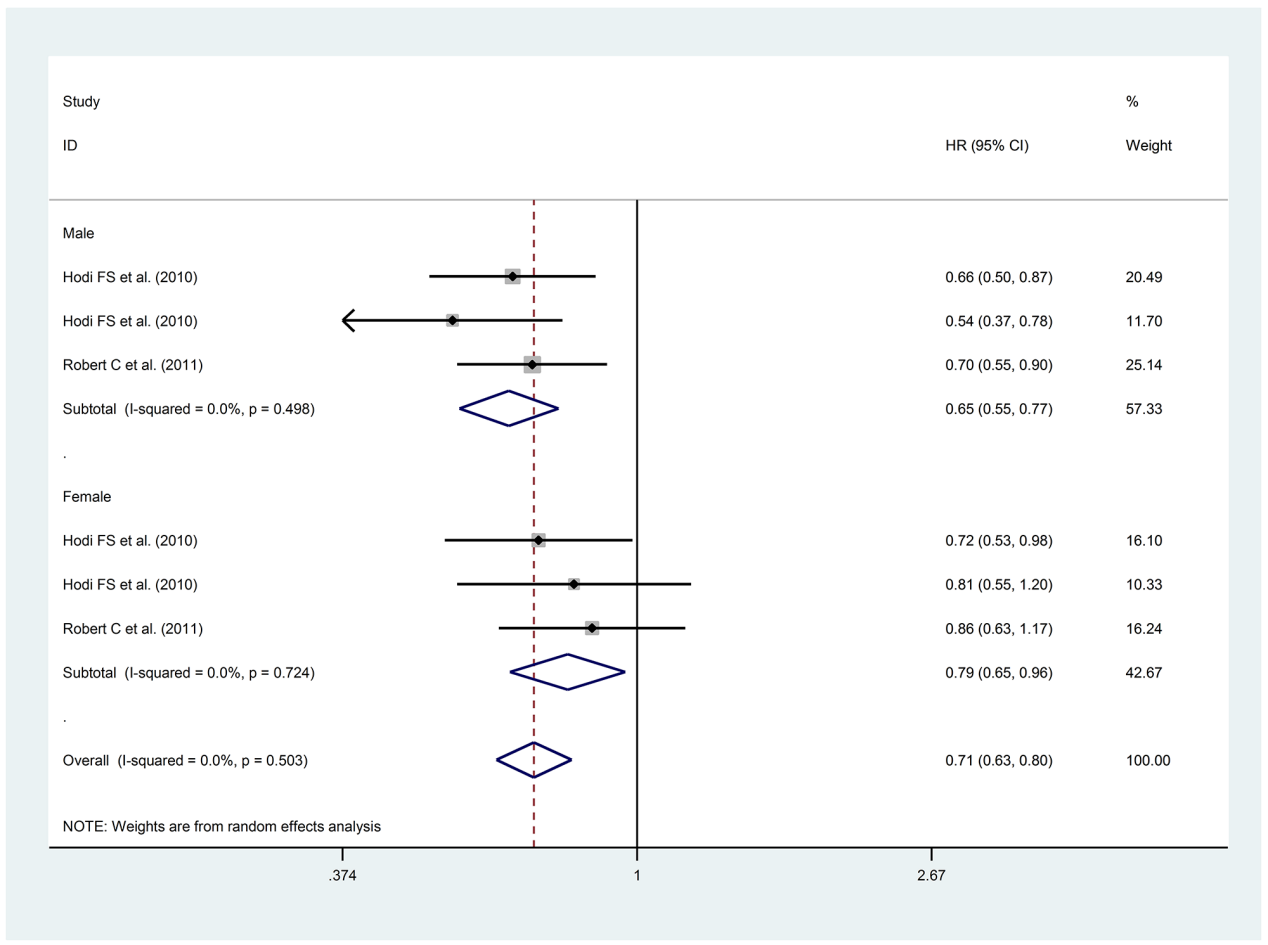
Forest Plot for OS with anti-CTLA-4

We performed a meta-analysis for OS with anti-CTLA-4 including 2 studies enrolling patients with melanoma treated with ipilimumab. (Figure 2) These trials enrolled 1,178 patients, 702 males (59.6%) and 476 females (40.4%). Overall, 480 males and 310 females received experimental treatment. [13, 16] The heterogeneity test between the two groups showed a benefit for males vs females (HR 0.65, 95% CI 0.55-0.77 *vs* HR 0.79, 95% CI 0.65-0.96, p 0.078).

**Figure 3:**
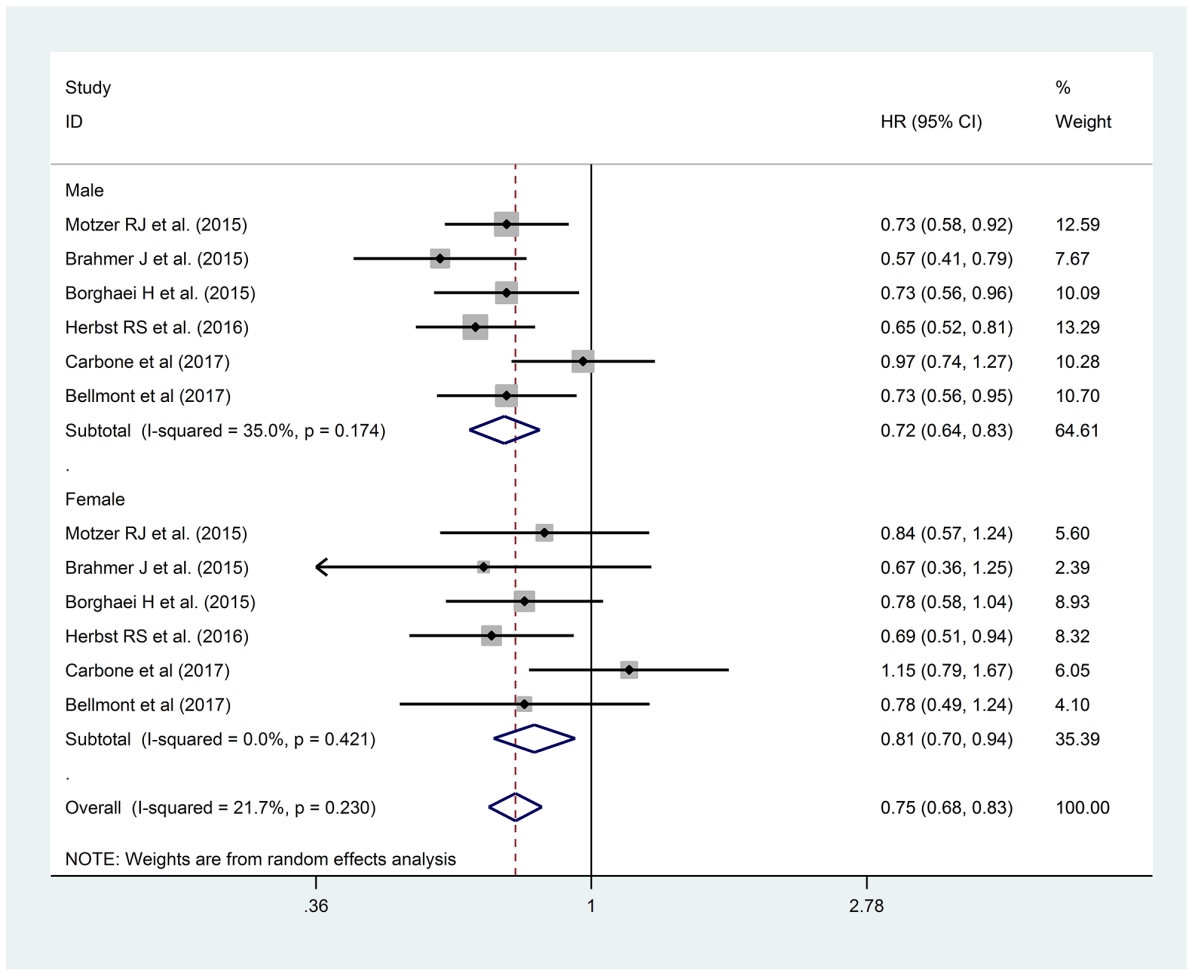
Forest Plot for OS with anti-PD-1

We considered 6 Clinical Trials with anti-PD-1 for OS. Overall we analyzed 3,792 patients, 2,514 males (66.3%) and 1,278 females (33.7%), 55.1% (1386) and 53.4% (683) of which received immunotherapy, respectively. [22, 26-30] In this analysis we observed a not statistically significant lower HR for males than females (HR 0.72, 95% CI 0.64-0.83 vs HR 0.81, 95% CI 0.70-0.94, p 0.285), as reported in Figure 3.

**Figure 4:**
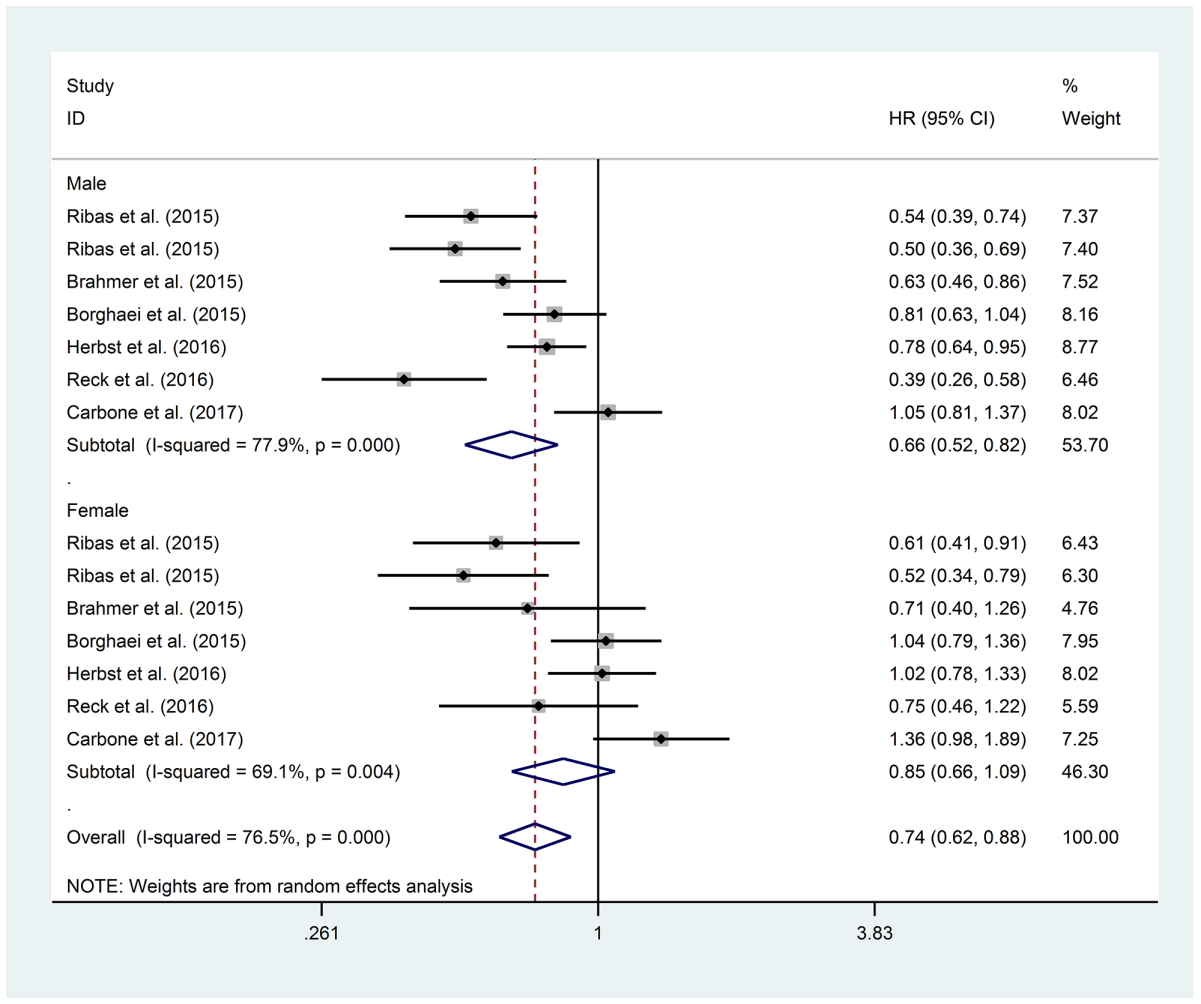
Forest Plot for PFS with anti-PD-1

Finally, we performed a meta-analysis for PFS, considering one Phase II and 5 Phase III Trials, as reported in Table 2. All the 6 studies randomized patients to receive anti-PD-1 (Pembrolizumab in 3 studies or Nivolumab in 3 studies). [22, 26-29, 32] The patients in the control arms received chemotherapy in first or second line, according to the trial. Only one study enrolled patients after failure of two lines of treatment. [32] Overall, we analyzed 3,274 patients, 2,007 males (61.3%) and 1,267 females (38.7%), 1,176 and 728 of which received experimental treatment, respectively. The heterogeneity test between sexes was not statistically significant, despite the HR lower in males group (HR 0.66, 95% CI 0.52-0.82 vs HR 0.85, 95% CI 0.66-1.09, p 0.158).(Figure 4) No PFS data differentiated by sex were available for anti-CTLA-4 or anti-PD-L1.

**Table 2:** Clinical trials selected for PFS

Author/year	Clinical trial	Cancer	Treatment	N pts	M	F	PFS HR	PFS M HR	PFS M range	PFS F HR	PFS F range
Ribas et al. 2015 [32]	Phase II	Melanoma St. IV	Pembrolizumab 2 mg/kg	180	104	76	0.57(0.45-0.73)	0.54	0.39-0.74	0.61	0.41-0.92
			Pembrolizumab 10 mg/kg	181	109	72	0.50(0.39-0.64)	0.50	0.36-0.68	0.52	0.34-0.78
			Chemotherapy	179	114	65					
Brahmer et al. 2015 [26]	Phase III	NSCLC St. IIIB/IV	Nivolumab 3 mg/kg	135	11	24	0.59(0.44-0.79)	0.57	0.41-0.78	0.67	0.36-1.25
			Docetaxel	137	97	40					
Borghaei et al. 2015 [27]	Phase III	NSCLC ADK St. IIIB/IV	Nivolumab 3 mg/kg	292	151	141	0.92(0.77-1.1)	0.81	0.63-1.04	1.04	0.80-1.37
			Docetaxel	290	168	122					
Carbone et al. 2017 [28]	Phase III	NSCLC St. IV/recurent	Nivolumab 3 mg/kg	271	184	87	1.15(0.91-1.45)	1.05	0.81-1.37	1.36	0.98-1.90
			Chemotherapy	270	148	122					
Herbst et al. 2016 [22]	Phase II/III	NCSLC St. IV	Pembrolizumab 2 mg/kg	345	212	133	0.88(0.74-1.05)	0.78	0.64-0.94	1.02	0.78-1.32
			Pembrolizumab 10 mg/kg	346	213	133	0.79(0.66-0.94)				
			Docetaxel	343	209	134					
Reck et al. 2016 [23]	Phase III	NSCLC St. IV	Pembrolizumab 200 mg	154	92	62	0.50(0.37-0.68)	0.39	0.26-0.58	0.75	0.46-1.21
			Chemotherapy	151	95	56					

## DISCUSSION

Males and females differ in the immune response to infections and vaccines and in the predisposition to develop autoimmune diseases. [4].

The observation that not only hormonal factors but also genetic, and environmental mediators are involved in sex-based immunological differences lead to hypothesize that different outcome during immunotherapy could depend on the patient’s sex.

The aim of our study is to analyze differences in the response to ICIs treatment in both sexes.

Interestingly in our study we observed a better OS associated to the anti-CTLA4 treatment in males compared to females. As far as anti-PD-1 treatment is concerned, despite the sample size and the number of clinical trials evaluated considerably wider than those for the anti CTLA-4 analysis, statistical significance has not been achieved.

Moreover we observed lower HR in males compared to women both for OS and PFS for anti-PD-1 treatments. Instead for anti PD-L1 antibodies it was not possible to perform a meta-analysis since in literature only one study presented HR for females and males.

It has been demonstrated that females have an immune system that acts predominantly by T helper (CD4+) response, in particular with a humoral response in various species and in cell culture systems in presence of high levels of estrogen and progesterone. [4] Hormone receptors are present in many cells of immune system; especially the estrogen receptor is expressed in lymphocytes, macrophages and dendritic cells, while progesterone receptor is present, also in the natural killer cells. [4, 51, 52] Conversely, males’ immune system mainly acts through a cytotoxic action, having at baseline a higher number of T CD8 + lymphocytes and a lower CD4 + / CD8 + ratio than females. [4] This last observation could results in a stronger CD8+ response against tumor and in a better sensibility to ICIs.

Furthermore it is important to underline that many genes involved in the immune response are located on the X chromosome and therefore they showed a higher expression in females. These genes encode for receptors belonging to the class of PRRs, as TLR4 and TLR7, involved in a humoral response, some receptors for interleukins and some transcription factors such as FOXP3 that acts as negative regulator. [5, 53].

Interestingly Griesbeck demonstrated, in a murine model, that the production of IFN-alfa after TRL7 stimulations was higher in female dendritic cells than males, suggesting a crucial role of IRF5 gene. [54].

Indeed, several genes, whose function is implicated in immune system activity, have in their promoter an estrogen response element (ERE), such the IRF5 gene, which encodes for the IFN regulatory factor 5. [54, 55] IFNγ gene presents also an ERE on its promoter, with a transcription directly regulated by estrogens. [4] IFNγ consequently promotes, through a IRF1 mediated mechanism, the expression of CD-274 gene, coding for the PD-L1, and of IDO gene, encoding for indoleamine-2,3-dioxygenase, an enzyme that transform tryptophan into kynurenine, leading to an immunosuppressive tumor microenvironment in which the T cells’ proliferation and activity are inhibited. [56-58].

Several studies demonstrated that Treg cells express high immune inhibitory receptors, such as CTLA-4 and PD-1. Paradoxically, on Treg cells the binding of these receptors with the ligand leads to an increase in the activation of the Treg cells themselves, with consequent inhibitory activity on T lymphocytes. [59] As it has been observed in different preclinical and clinical studies, the use of anti-CTLA-4 antibodies could induce the depletion of Treg cells. [60, 61] As Treg cells are present at higher levels in males, this mechanism could affect the different response to anti-CTLA-4 in the two sexes. [4] As far as PD-1, despite having a Treg cell activation function similar to that described for CTLA-4, an inhibitory action on Treg cells has not been reported in literature.

The inhibitory activity of anti CTLA-4 on Treg cells also occurs at the gastrointestinal tract, with dysregulation of their immunomodulatory function and consequent widespread inflammation, particularly at the colon level. [62, 63] This is certainly the basis of the gastrointestinal toxicity mechanism but also responsible for the modification of the intestinal flora. The function of microbiota is relevant for the modulation of the immune system; moreover it’s affected by gender and diet differences, and it is also involved in immune-related toxicity mechanisms during treatement. [64] The effect of the microbiota may also affect the different efficacy observed according to the sex, and among different drugs with different mechanism of action.

Furthermore the differences of PFS and OS HR between anti-CTLA-4 and anti-PD-1 could depend on their different action’s mechanism. Anti-CTLA-4 and anti PD-1 act in different point of cancer- immune cycle (in priming phase and killing cancer cells phase respectively): while anti CTLA4 enhances early T cell activation, anti-PD-1 reverse the exhausted state of existing effector T cells. Thus the different activation status of antigen-specific T cells in males and females could influences the response not only to monotherapy but mostly to combination of ICIs.

Moreover, we have to consider also the differences in gender and not only in sex. The environmental estrogens could have an impact on immune response such as xenoestrogens added in cosmetic or hormone replacement therapy and oral contraceptives.

Our work presents some weakness and limitations depending on the trials’ heterogeneity, on the different cancer type considered that could have different immunogenicity and on the absence of information about hormonal status and on PD-L1 status according to sex.

Furthermore, some studies enrolled patients according to biomarkers expression at different cut-off, for example PD-L1 for Pembrolizumab trials or for first-line Nivolumab, while for anti-CTLA-4 trials none biomarker was used as inclusion criteria. Considering the previously discussed sex related implications on PD-L1 expression, this heterogeneity between trials could be considered as a bias of our meta-analysis.

Moreover, this is not an individual patients’ data meta-analysis, leading to exclusion of many literature data. We also excluded trials with control arm treated with ICIs and trials studying combination immunotherapy, even if it could be interesting to analyze the impact of the double block of immune target in the two sexes. However, we decided to include only Phase II-III trials and to exclude trials with combination of ICIs to reduce the possible bias.

These observations need to be confirmed by further studies, in particular by direct comparison of males and females on a homogeneous and numerically larger sample, with an analysis of lymphocyte subpopulations, of expression of different markers on the surface of immune cells, of mutational burden, with the dosage of cytokines and antibodies during therapy, with an accurate study of the microbiota and studying the possible associations with immune related adverse events (irAE).

In conclusion, the differences in immune response between males and females could be relevant to determine the response and the resistance to ICIs and to identify new immunological targets in order to draw studies exploring novels combinations of immunotherapy agents.

## MATERIALS AND METHODS

### Data retrieval

We conducted a meta-analysis of Clinical Trials including treatment with immunotherapy (anti-CTLA-4, anti-PD1, anti-PD-L1), which data were published up to June 2017. The databases of PubMed, www.clinicaltrials.gov and the American Society of Clinical Oncology (ASCO) University Meeting was searched for relevant publications about lung (NSCLC), renal cells (RCC), head and neck, urothelial cancers and melanoma. Furthermore, we completed the data retrieval with a manual search between the references of the previously selected articles. The selection of items was restricted to phase II and phase III trials.

### Data extraction

Three independent reviewers selected and analyzed all the articles. The following data were extracted: type of study; type of cancer; stage of disease; arms of treatment; number and sex of patients for each arm of treatment; PFS and OS expressed as HR for the whole population and differentiated by sex. The abstracts were excluded for lack of subgroups’ analysis. Similarly, the articles where subgroup analysis differentiated by was not reported were excluded from the final statistical analysis. The studies in which the control arm received ICIs where excluded too.

### Statistical analysis

Hazard Ratios (HR) with 95% confidence intervals (CIs) for PFS and OS were extracted from papers. Subgroups random-effects meta-analysis (inverse-variance weighted method) were performed to evaluate differences in treatment effects according to patients’ sex. In all figures the derived results are reported as conventional meta-analysis forest plots, with a HR<1.0 indicating better outcome in the experimental arm. All the analysis were accomplished using Stata/SE14.0 (Stata Corp, College Station, TX, USA).
